# Checklist of the family Syrphidae (Diptera) of Finland

**DOI:** 10.3897/zookeys.441.7251

**Published:** 2014-09-19

**Authors:** Antti Haarto, Sakari Kerppola

**Affiliations:** 1 Zoological Museum, Section of Biodiversity and Environmental Science, Department of Biology, FI-20014 University of Turku, Finland; 2 Hiihtomäentie 44 A 6, FI-00800 Helsinki, Finland

**Keywords:** Checklist, Finland, Diptera, Syrphidae, faunistics

## Abstract

A checklist of the Syrphidae (Diptera) recorded from Finland. Three species of Syrphidae, *Platycheirus modestus* Ide, 1926, *Cheilosia barovskii* (Stackelberg, 1930) and *Mallota tricolor* Loew, 1871, are published as new to the Finnish fauna. *Platycheirus modestus* is also new to the Palaearctic.

## Introduction

Adults of Syrphidae (hoverflies or flower flies) are mimics of bees, bumblebees, wasps and sawflies. Syrphid flies are active visitors of flowers for nectar and pollen and therefore they are important pollinators. The syrphid adults are excellent fliers and often seen hovering.

The larvae of hoverflies exploit a wide range of food sources. The majority of the species are predators and a large number of them feed on aphids. Almost half of the syrphid species are phytophagous or saprophagous. The larvae of phytophagous species tunnel in roots and stems of plants and some mine in leaves, but this rarely causes significant economic damage. Aquatic larvae are saprophagous in decaying organic material and feed on detritus or filter bacteria. Also larvae living in dead wood and in sap runs probably filter bacteria from the decaying mass. (Marshall 2012, Van Veen 2004).

Only a few active Diptera researchers, Richard Frey, Wolter Hellén and Erkki Kanervo, studied the fauna of Finnish syrphids from the 1910s to the 1960s. They created a very good base for the knowledge of the Finnish syrphid fauna. Hackman (1980) published a list of the Finnish flies including 270 species of hoverflies. The beginning of the 2000s was an active period for faunistic research of the hoverfly fauna and the number of recorded hoverfly species had increased to 347 as the book on Finnish hoverflies (Haarto and Kerppola 2007a) was published. After that the knowledge has still been increasing thanks to working on the taxonomy and faunistics of the Finnish hoverfly fauna so that the distribution can be considered as well-known for over 90 per cent of the Finnish hoverfly species (Kahanpää 2010).

The Finnish hoverfly species were last listed by Kerppola (2013) with additions and omissions of species and with some nomenclature changes.

Identification: The Finnish syrphid fauna is best covered for identification by Haarto and Kerppola (2007a) and Bartsch (2009) although some species are missing in these books. Most of the species can be identified by external characters but some species require male genitalic characters for reliable determination.

**Number of species:**

World: 6015 species (Pape et al. 2011)

Europe: 830 species

Finland: 362 species

Faunistic knowledge level in Finland: good

## Checklist

suborder Brachycera Macquart, 1834

clade Eremoneura Lameere, 1906

clade Aschiza Becher, 1882

superfamily Syrphoidea Latreille, 1802

**SYRPHIDAE** Latreille, 1802

SYRPHINAE Latreille, 1802

tribe Bacchini Bigot, 1883

***BACCHA*** Fabricius, 1805

*Baccha elongata* (Fabricius, 1775)

= *obscuripennis* Meigen, 1822

tribe Melanostomini Williston, 1885

***MELANOSTOMA*** Schiner, 1860

*Melanostoma dubium* (Zetterstedt, 1837)

= *freyi* (Hellén, 1950)

*Melanostoma mellinum* (Linnaeus, 1758)

*Melanostoma scalare* (Fabricius, 1794)

***PLATYCHEIRUS*** Le Peletier & Serville, 1828

**sg. *Platycheirus*** Le Peletier & Serville, 1828

*Platycheirus aeratus* Coquillet, 1900

= *angustitarsis* Kanervo, 1934

*Platycheirus albimanus* (Fabricius, 1781)

*Platycheirus ambiguus* (Fallén, 1817)

*Platycheirus amplus* Curran, 1927

*Platycheirus angustatus* (Zetterstedt, 1843)

*Platycheirus aurolateralis* (Stubbs, 2002)

*Platycheirus brunnifrons* Nielsen, 2004

*Platycheirus carinatus* (Curran, 1927)

? = *hirtipes* Kanervo, 1938

*Platycheirus clypeatus* (Meigen, 1822)

*Platycheirus discimanus* Loew, 1871

*Platycheirus europaeus* Goeldlin, Maibach & Speight, 1990

*Platycheirus fulviventris* (Macquart, 1829)

*Platycheirus goeldlini* Nielsen, 2004

*Platycheirus groenlandicus* Curran, 1927

= *boreomontanus* Nielsen, 1981

= *monticolus* Nielsen, 1972 preocc.

*Platycheirus holarcticus* Vockeroth, 1990

*Platycheirus hyperboreus* (Staeger, 1844)

*Platycheirus immarginatus* (Zetterstedt, 1849)

*Platycheirus jaerensis* Nielsen, 1971

*Platycheirus kittilaensis* Dušek & Láska, 1982

= *complicatus* misid.

*Platycheirus laskai* Nielsen, 1999

*Platycheirus latimanus* (Wahlberg, 1844)

*Platycheirus lundbecki* (Collin, 1931)

*Platycheirus magadanensis* Mutin, 1999

*Platycheirus manicatus* (Meigen, 1822)

*Platycheirus modestus* Ide, 1926

*Platycheirus nielseni* Vockeroth, 1990

*Platycheirus nigrofemoratus* Kanervo, 1934

*Platycheirus occultus* Goeldlin, Maibach & Speight, 1990

*Platycheirus parmatus* Rondani, 1857

*Platycheirus peltatus* (Meigen, 1822)

*Platycheirus perpallidus* Verral, 1901

*Platycheirus podagratus* (Zetterstedt, 1838)

*Platycheirus ramsarensis* Goeldlin, Maibach & Speight, 1990

*Platycheirus scambus* (Staeger, 1843)

*Platycheirus scutatus* (Meigen, 1822)

*Platycheirus splendidus* Rotheray, 1998

*Platycheirus sticticus* (Meigen, 1822)

*Platycheirus subordinatus* Becker, 1915

*Platycheirus tarsalis* (Schummel, 1836)

*Platycheirus transfugus* (Zetterstedt, 1838)

*Platycheirus urakawensis* (Matsumura, 1919)

*Platycheirus varipes* Curran, 1923

**sg. *Pyrophaena*** Schiner, 1860

*Platycheirus granditarsus* (Forster, 1771)

*Platycheirus rosarum* (Fabricius, 1787)

***XANTHANDRUS*** Verrall, 1901

*Xanthandrus comtus* (Harris, 1780)

tribe Paragini Glumac, 1961

***PARAGUS*** Latreille, 1804

*Paragus constrictus* Šimić, 1986

*Paragus finitimus* Goeldlin, 1971

*Paragus haemorrhous* Meigen, 1822

= *sigillatus* Curtis, 1836

*Paragus pecchiolii* Rondani, 1857

= *majoranae* auct. nec Rondani, 1857

= *albifrons* misid.

*Paragus tibialis* (Fallén, 1817)

tribe Chrysotoxini Newman, 1834

***CHRYSOTOXUM*** Meigen, 1803

*Chrysotoxum arcuatum* (Linnaeus, 1758)

= *fasciatum* (Müller, 1764)

*Chrysotoxum bicinctum* (Linnaeus, 1758)

*Chrysotoxum cautum* (Harris, 1776)

*Chrysotoxum fasciolatum* (De Geer, 1776)

*Chrysotoxum festivum* (Linnaeus, 1758)

= *arcuatum* auct. nec (Linnaeus, 1758)

*Chrysotoxum octomaculatum* Curtis, 1937

= *elegans* misid.

*Chrysotoxum vernale* Loew, 1841

tribe Syrphini Latreille, 1802

***DASYSYRPHUS*** Enderlein, 1838

*Dasysyrphus albostriatus* (Fallén, 1817)

*Dasysyrphus friuliensis* (van der Goot, 1960)

= *claviger* (Frey, 1930)

= *carpathicus* Stys & Moucha, 1962

*Dasysyrphus hilaris* (Zetterstedt, 1843)

*Dasysyrphus nigricornis* (Verrall, 1873)

= *obscuratus* (Ringdahl, 1928)

*Dasysyrphus pauxillus* (Williston, 1887)

*Dasysyrphus pinastri* (De Geer, 1776)

= *lunulatus* (Meigen, 1822)

*Dasysyrphus postclaviger* Stys & Moucha, 1962

= *claviger* (Frey, 1930) preocc.

*Dasysyrphus tricinctus* (Fallén, 1817)

*Dasysyrphus venustus* (Meigen, 1822)

= *arcuatus* (Fallén, 1817)

***DIDEA*** Macquart, 1834

*Didea alneti* (Fallén, 1817)

*Didea fasciata* Macquart, 1834

*Didea intermedia* Loew, 1854

***DOROS*** Meigen, 1803

*Doros profuges* (Harris, 1780)

= *conopseus* auct. nec (Linnaeus, 1758)

***EPISTROPHE*** Walker, 1852

*Epistrophe annulitarsis* Stackelberg, 1918

*Epistrophe cryptica* Doczkal & Schmid, 1994

*Epistrophe diaphana* (Zetterstedt, 1843)

*Epistrophe eligans* (Harris, 1780)

= *bifasciata* (Fabricius, 1794) preocc.

*Epistrophe flava* Doczkal & Schmid, 1994

= *melanostomoides* misid.

*Epistrophe grossulariae* (Meigen, 1822)

*Epistrophe melanostoma* (Zetterstedt, 1843)

= *melanostomoides* Strobl, 1910

*Epistrophe nitidicollis* (Meigen, 1822)

*Epistrophe obscuripes* (Strobl, 1910)

= *similis* Doczkal & Schmid, 1994

*Epistrophe ochrostoma* (Zetterstedt, 1849)

*Epistrophe olgae* Mutin, 1990

***EPISTROPHELLA*** Dušek & Láska, 1967

*Epistrophella euchroma* (Kowarz, 1885)

***EPISYRPHUS*** Matsumura & Adachi, 1917

*Episyrphus balteatus* (De Geer, 1776)

***ERIOZONA*** Schiner, 1860

*Eriozona syrphoides* (Fallén, 1817)

***EUPEODES*** Osten Sacken, 1877

= ***Metasyrphus*** Matsumura, 1917

*Eupeodes abiskoensis* (Dušek & Láska, 1973)

*Eupeodes biciki* (Nielsen, 2003)

*Eupeodes bucculatus* (Rondani, 1857)

= *latilunulatus* (Collin, 1931)

*Eupeodes corollae* (Fabricius, 1794)

*Eupeodes curtus* (Hine, 1922)

*Eupeodes duseki* Mazánek, Láska & Bicik, 1999

*Eupeodes goeldlini* Mazánek, Láska & Bicik, 1999

*Eupeodes latifasciatus* (Macquart, 1829)

*Eupeodes lundbecki* (Soot-Ryen, 1946)

*Eupeodes luniger* (Meigen, 1822)

*Eupeodes nielseni* (Dušek & Láska, 1976)

*Eupeodes nitens* (Zetterstedt, 1843)

*Eupeodes punctifer* (Frey, 1934)

= *borealis* misid.

*Eupeodes tirolensis* (Dušek & Láska, 1973)

***FAGISYRPHUS*** Dušek & Láska, 1967

*Fagisyrphus cinctus* (Fallén, 1817)

***LAPPOSYRPHUS*** Dušek & Láska, 1967

*Lapposyrphus lapponicus* (Zetterstedt, 1838)

***LEUCOZONA*** Schiner, 1860

**sg. *Leucozona*** Schiner, 1860

*Leucozona inopinata* Doczkal, 2000

*Leucozona lucorum* (Linnaeus, 1758)

**sg. *Ischyrosyrphus*** Bigot, 1882

*Leucozona glaucia* (Linnaeus, 1758)

*Leucozona laternaria* (Müller, 1776)

***MEGASYRPHUS*** Duššek & Láska, 1967

*Megasyrphus erraticus* (Linnaeus, 1758)

= *annulipes* (Zetterstedt, 1838)

***MELANGYNA*** Verrall, 1901

*Melangyna arctica* (Zetterstedt, 1838)

*Melangyna barbifrons* (Fallén, 1817)

*Melangyna coei* Nielsen, 1971

*Melangyna compositarum* (Verrall, 1873)

*Melangyna lasiophthalma* (Zetterstedt, 1843)

*Melangyna lucifera* Nielsen, 1980

*Melangyna quadrimaculata* (Verrall, 1873)

*Melangyna umbellatarum* (Fabricius, 1794)

***MELIGRAMMA*** Frey, 1946

*Meligramma guttata* (Fallén, 1817)

*Meligramma triangulifera* (Zetterstedt, 1843)

***MELISCAEVA*** Frey, 1946

*Meliscaeva auricollis* (Meigen, 1822)

*Meliscaeva cinctella* (Zetterstedt, 1843)

***PARASYRPHUS*** Matsumura, 1917

*Parasyrphus annulatus* (Zetterstedt, 1838)

*Parasyrphus groenlandicus* (Nielsen, 1910)

*Parasyrphus lineolus* (Zetterstedt, 1843)

*Parasyrphus macularis* (Zetterstedt, 1843)

*Parasyrphus malinellus* (Collin, 1952)

*Parasyrphus nigritarsis* (Zetterstedt, 1843)

*Parasyrphus proximus* Mutin, 1990

*Parasyrphus punctulatus* (Verrall, 1873)

*Parasyrphus tarsatus* (Zetterstedt, 1838)

= *dryadis* (Holmgren, 1869)

*Parasyrphus vittiger* (Zetterstedt, 1843)

***SCAEVA*** Fabricius, 1805

*Scaeva pyrastri* (Linnaeus, 1758)

*Scaeva selenitica* (Meigen, 1822)

***SPHAEROPHORIA*** Lepeletier & Serville, 1828

*Sphaerophoria abbreviata* Zetterstedt, 1859

*Sphaerophoria bankowskae* Goeldlin, 1989

*Sphaerophoria batava* Goeldlin, 1974

*Sphaerophoria boreoalpina* Goeldlin, 1989

*Sphaerophoria chongjini* Bankowska, 1964

*Sphaerophoria fatarum* Goeldlin, 1989

*Sphaerophoria interrupta* (Fabricius, 1805)

= *menthastri* (Linnaeus, 1758)

*Sphaerophoria kaa* Violovitsh, 1960

*Sphaerophoria laurae* Goeldlin, 1989

*Sphaerophoria loewi* Zetterstedt, 1843

*Sphaerophoria pallidula* Mutin, 1999

*Sphaerophoria philantha* (Meigen, 1822)

= *sarmatica* Bankowska, 1964

*Sphaerophoria rueppelli* (Wiedemann, 1830)

*Sphaerophoria scripta* (Linnaeus, 1758)

*Sphaerophoria taeniata* (Meigen, 1822)

*Sphaerophoria virgata* Goeldlin, 1974

***SYRPHUS*** Fabricius, 1775

*Syrphus admirandus* Goeldlin, 1996

*Syrphus attenuatus* Hine, 1922

= *pilisquamus* Ringdahl, 1928

*Syrphus ribesii* (Linnaeus, 1758)

= *brevicinctus* Kanervo, 1938

*Syrphus sexmaculatus* Zetterstedt, 1838

*Syrphus torvus* Osten Sacken, 1875

*Syrphus vitripennis* Meigen, 1822

= *rectus bretoletensis* Goeldlin, 1996

***XANTHOGRAMMA*** Schiner, 1860

*Xanthogramma citrofasciatum* (De Geer, 1776)

= *festivum* auct. nec (Linnaeus, 1758)

*Xanthogramma pedissequum* (Harris, 1776)

*Xanthogramma stackelbergi* Violovitsh, 1975

MILESIINAE Rondani, 1845

tribe Cerioidini Wahlgren, 1909

***CERIANA*** Rafinesque, 1815

*Ceriana conopsoides* (Linnaeus, 1758)

***SPHIXIMORPHA*** Rondani, 1850

*Sphiximorpha subsessilis* (Illiger in Rossi, 1807)

tribe Cheilosini Williston, 1885

***CHEILOSIA*** Meigen, 1822

*Cheilosia alba* Vujić & Claussen, 2000

*Cheilosia albipila* Meigen, 1838

*Cheilosia albitarsis* (Meigen, 1822)

*Cheilosia alpina* (Zetterstedt, 1838)

*Cheilosia angustigenis* Becker, 1894

*Cheilosia barbata* Loew, 1857

= *honesta* Rondani, 1868

*Cheilosia barovskii* (Stackelberg, 1930)

*Cheilosia carbonaria* Egger, 1860

*Cheilosia chrysocoma* (Meigen, 1822)

*Cheilosia cynocephala* Loew, 1840

*Cheilosia flavipes* (Panzer, 1798)

*Cheilosia flavissima* Becker, 1894

= *pallipes* auct. nec Loew, 1863

*Cheilosia fraterna* (Meigen, 1830)

*Cheilosia frontalis* Loew, 1857

*Cheilosia gigantea* (Zetterstedt, 1838)

= *gracilis* Hellén, 1914

*Cheilosia grossa* (Fallén, 1817)

*Cheilosia illustrata* (Harris, 1780)

*Cheilosia impressa* Loew, 1840

*Cheilosia ingerae* Nielsen & Claussen, 2001

*Cheilosia lasiopa* Kowarz, 1885

= *honesta* auct. nec Rondani, 1868

*Cheilosia latifrons* (Zetterstedt, 1843)

= *intonsa* Loew, 1857

*Cheilosia longula* (Zetterstedt, 1838)

= *plumulifera* Loew, 1857

= *soror* misid.

*Cheilosia melanopa* (Zetterstedt, 1843)

*Cheilosia morio* (Zetterstedt, 1838)

*Cheilosia mutabilis* (Fallén, 1817)

= *ruralis* (Meigen, 1822)

*Cheilosia naruska* Haarto & Kerppola, 2007

*Cheilosia nebulosa* Verrall, 1871

= *langhofferi* Becker, 1894

*Cheilosia nigripes* (Meigen, 1822)

*Cheilosia pagana* (Meigen, 1822)

*Cheilosia proxima* (Zetterstedt, 1843)

*Cheilosia psilophthalma* Becker, 1894

*Cheilosia pubera* (Zetterstedt, 1838)

*Cheilosia reniformis* Hellén, 1930

*Cheilosia rufimana* Becker, 1894

*Cheilosia sahlbergi* Becker, 1894

*Cheilosia scutellata* (Fallén, 1817)

*Cheilosia semifasciata* Becker, 1894

*Cheilosia sootryeni* Nielsen, 1970

*Cheilosia urbana* (Meigen, 1822)

= *ruralis* (Meigen, 1822)

= *praecox* (Zetterstedt, 1843)

= *globulipes* Becker, 1894

*Cheilosia uviformis* Becker, 1894

= *argentifrons* Hellén, 1914

*Cheilosia variabilis* (Panzer, 1798)

*Cheilosia velutina* Loew, 1840

*Cheilosia vernalis* (Fallén, 1817)

= *rotundicornis* Hellén, 1914

= *rotundiventris* Becker, 1894

= *ruficollis* Becker, 1894

*Cheilosia vicina* (Zetterstedt, 1849)

= *nasutula* Becker, 1894

*Cheilosia vulpina* (Meigen, 1822)

*Cheilosia* sp. A in Haarto & Kerppola, 2007

***FERDINANDEA*** Rondani, 1844

*Ferdinandea cuprea* (Scopoli, 1763)

***RHINGIA*** Scopoli, 1763

*Rhingia borealis* Ringdahl, 1928

= *austriaca* auct. nec Meigen, 1830

*Rhingia campestris* Meigen, 1822

tribe Chrysogasterini Shannon, 1922

***BRACHYOPA*** Meigen, 1822

*Brachyopa cinerea* Wahlberg, 1844

*Brachyopa dorsata* Zetterstedt, 1837

*Brachyopa obscura* Thompson & Torp, 1982

*Brachyopa pilosa* Collin, 1939

= *bicolor* misid.

*Brachyopa testacea* (Fallén, 1817)

= *conica* misid.

*Brachyopa vittata* Zetterstedt, 1843

*Brachyopa zhelochovtsevi* Mutin, 1998

***CHRYSOGASTER*** Meigen, 1803

*Chrysogaster coemiteriorum* (Linnaeus, 1758)

= *cemiteriorum* emend.

= *chalybeata* Meigen, 1822

*Chrysogaster solstitialis* (Fallén, 1817)

*Chrysogaster virescens* Loew, 1854

***CHRYSOSYRPHUS*** Sedman, 1965

= ***Helleniola*** Stackelberg, 1965

*Chrysosyrphus nasutus* (Zetterstedt, 1838)

*Chrysosyrphus niger* (Zetterstedt, 1843)

= *Myolepta nigra* mistake

***HAMMERSCHMIDTIA*** Schummel, 1834

*Hammerschmidtia ferruginea* (Fallén, 1817)

*Hammerschmidtia ingrica* Stackelberg, 1952

***LEJOGASTER*** Rondani, 1857

*Lejogaster metallina* (Fabricius, 1781)

*Lejogaster tarsata* (Meigen, 1822)

= *splendida* (Meigen, 1822)

***MELANOGASTER*** Rondani, 1857

*Melanogaster aerosa* (Loew, 1843)

= *macquarti* (Loew, 1843)

= *viduata* misid.

*Melanogaster parumplicata* (Loew, 1840)

***NEOASCIA*** Williston, 1886

**sg. *Neoascia*** Williston, 1886

*Neoascia podagrica* (Fabricius, 1775)

*Neoascia tenur* (Harris, 1780)

= *dispar* (Meigen, 1822)

= *lapponica* Kanervo, 1934

= *splendida* Kanervo, 1934

**sg. *Neoasciella*** Stackelberg, 1970

*Neoascia geniculata* (Meigen, 1822)

*Neoascia interrupta* (Meigen, 1822)

*Neoascia meticulosa* (Scopoli, 1763)

= *aenea* (Meigen, 1822)

*Neoascia obliqua* Coe, 1940

*Neoascia subchalybea* Curran, 1925

= *petsamoensis* Kanervo, 1934

***ORTHONEVRA*** Macquart, 1829

*Orthonevra elegans* (Meigen, 1822)

*Orthonevra erythrogona* (Malm, 1863)

*Orthonevra geniculata* (Meigen, 1830)

= *linnaniemii* Kanervo, 1934

*Orthonevra intermedia* Lundbeck, 1916

= *rossica* Stackelberg, 1953

*Orthonevra nobilis* (Fallén, 1817)

*Orthonevra plumbago* (Loew, 1840)

= *brevicornis* misid.

*Orthonevra stackelbergi* Thompson & Torp, 1982

= *intermedia* misid.

***SPHEGINA*** Meigen, 1822

**sg. *Sphegina*** Meigen, 1822

*Sphegina clunipes* (Fallén, 1816)

*Sphegina elegans* Schummel, 1843

= *kimakowiczi* misid.

*Sphegina montana* Becker, 1921

= *violovitshi* misid.

= *verecunda* misid.

*Sphegina spheginea* (Zetterstedt, 1838)

**sg. *Asiosphegina*** Stackelberg, 1975

*Sphegina sibirica* Stackelberg, 1953

tribe Eristalini Newman, 1834

***ANASIMYIA*** Schiner, 1864

= ***Eurimyia*** Bigot, 1883

*Anasimyia contracta* Claussen & Torp, 1980

*Anasimyia interpuncta* (Harris, 1776)

*Anasimyia lineata* (Fabricius, 1787)

*Anasimyia lunulata* (Meigen, 1822)

*Anasimyia transfuga* (Linnaeus, 1758)

***ERISTALINUS*** Rondani, 1845

*Eristalinus aeneus* (Scopoli, 1763)

*Eristalinus sepulchralis* (Linnaeus, 1758)

***ERISTALIS*** Latreille, 1804

*Eristalis abusiva* Collin, 1931

*Eristalis alpina* (Panzer, 1798)

*Eristalis anthophorina* (Fallén, 1817)

*Eristalis arbustorum* (Linnaeus, 1758)

= *nemorum* auct. nec (Linnaeus, 1758)

*Eristalis cryptarum* (Fabricius, 1794)

*Eristalis fratercula* Zetterstedt, 1838

= *vallei* Kanervo, 1934

*Eristalis gomojunovae* Violovitsh, 1977

= *fratercula* auct. nec Zetterstedt, 1838

*Eristalis hirta* Loew, 1866

= *Eristalis tundrarum* Frey, 1932

*Eristalis horticola* (De Geer, 1776)

= *lineata* (Harris, 1776)

*Eristalis intricaria* (Linnaeus, 1758)

*Eristalis nemorum* (Linnaeus, 1758)

= *interrupta* (Poda, 1761)

*Eristalis obscura* Loew, 1866

= *pseudorupium* Kanervo, 1938

= *vitripennis* Strobl, 1893

*Eristalis oestracea* (Linnaeus, 1758)

*Eristalis pertinax* (Scopoli, 1763)

*Eristalis picea* (Fallén, 1817)

*Eristalis rupium* (Fabricius, 1805)

*Eristalis similis* (Fallén, 1817)

= *pratorum* (Meigen, 1822)

*Eristalis tenax* (Linnaeus, 1758)

***HELOPHILUS*** Meigen, 1822

*Helophilus affinis* Wahlberg, 1844

*Helophilus bottnicus* Wahlberg, 1844

*Helophilus groenlandicus* (Fabricius, 1780)

*Helophilus hybridus* Loew, 1846

*Helophilus lapponicus* Wahlberg, 1844

= *borealis* Staeger, 1845

*Helophilus pendulus* (Linnaeus, 1758)

*Helophilus trivittatus* (Fabricius, 1805)

= *parallelus* (Harris, 1776)

***MALLOTA*** Meigen, 1822

*Mallota megilliformis* (Fallén, 1817)

*Mallota tricolor* Loew, 1871

***MYATHROPA*** Rondani, 1845

*Myathropa florea* (Linnaeus, 1758)

***PARHELOPHILUS*** Girschner, 1897

*Parhelophilus consimilis* (Malm, 1863)

*Parhelophilus frutetorum* (Fabricius, 1775)

*Parhelophilus versicolor* (Fabricius, 1794)

tribe Eumerini Smirnov, 1924

***EUMERUS*** Meigen, 1822

*Eumerus flavitarsis* Zetterstedt, 1843

*Eumerus funeralis* Meigen, 1822

= *tuberculatus* Rondani, 1857

*Eumerus grandis* Meigen, 1822

= *annulatus* (Panzer, 1798)

*Eumerus ruficornis* Meigen, 1822

*Eumerus sabulonum* (Fallén, 1817)

*Eumerus strigatus* (Fallén, 1817)

tribe Merodontini Edwards, 1915

***MERODON*** Meigen, 1803

*Merodon equestris* (Fabricius, 1794)

tribe Pelecocerini Williston, 1887

***PELECOCERA*** Meigen, 1822

= ***Chamaesyrphus*** Mik, 1895

*Pelecocera caledonica* (Collin, 1940)

*Pelecocera lusitanica* (Mik, 1898)

*Pelecocera scaevoides* (Fallén, 1817)

*Pelecocera tricincta* Meigen, 1822

tribe Pipizini Williston, 1885

***CRYPTOPIPIZA*** Mutin, 1998

*Cryptopipiza notabila* (Violovitsh, 1985)

***HERINGIA*** Rondani, 1856

*Heringia heringi* (Zetterstedt, 1843)

***NEOCNEMODON*** Goffe, 1944

= ***Cnemodon*** Egger, 1865 preocc.

*Neocnemodon fulvimanus* (Zetterstedt, 1843)

*Neocnemodon larusi* (Vujić, 1999)

= *fulvimanus* auct. nec (Zetterstedt, 1843)

*Neocnemodon latitarsis* (Egger, 1865)

*Neocnemodon pubescens* (Delucchi & Pschorn-Walcher, 1955)

*Neocnemodon verrucula* (Collin, 1931)

*Neocnemodon vitripennis* (Meigen, 1822)

***PIPIZA*** Fallén, 1810

*Pipiza accola* Violovitsh, 1985

*Pipiza austriaca* Meigen, 1822

*Pipiza fasciata* Meigen, 1822

*Pipiza festiva* Meigen, 1822

*Pipiza lugubris* (Fabricius, 1775)

*Pipiza luteitarsis* Zetterstedt, 1843

*Pipiza noctiluca* (Linnaeus, 1758)

*Pipiza notata* Meigen, 1822

= *bimaculata* Meigen, 1822

*Pipiza quadrimaculata* (Panzer, 1804)

***PIPIZELLA*** Rondani, 1856

*Pipizella certa* Violovitsh, 1981

= *brevis* auct. nec Lucas, 1976

*Pipizella obscura* Van Steenis & Lucas, 2011

*Pipizella viduata* (Linnaeus, 1758)

= *virens* misid.

***TRICHOPSOMYIA*** Williston, 1888

*Trichopsomyia flavitarsis* (Meigen, 1822)

*Trichopsomyia joratensis* Goeldlin, 1997

***TRIGLYPHUS*** Loew, 1840

*Triglyphus primus* Loew, 1840

tribe Sericomyiini Rondani, 1845

***SERICOMYIA*** Meigen, 1803

*Sericomyia arctica* Schirmer, 1913

*Sericomyia jakutica* (Stackelberg, 1927)

*Sericomyia lappona* (Linnaeus, 1758)

*Sericomyia nigra* Portschinsky, 1872

*Sericomyia silentis* (Harris, 1776)

tribe Volucellini Newman, 1834

***VOLUCELLA*** Geoffroy, 1762

*Volucella bombylans* (Linnaeus, 1758)

*Volucella inanis* (Linnaeus, 1758)

*Volucella pellucens* (Linnaeus, 1758)

tribe Xylotini Bigot, 1883

***BLERA*** Billberg, 1820

*Blera eoa* (Stackelberg 1928)

*Blera fallax* (Linnaeus, 1758)

***BRACHYPALPOIDES*** Hippa, 1978

*Brachypalpoides lentus* (Meigen, 1822)

***BRACHYPALPUS*** Macquart, 1834

*Brachypalpus laphriformis* (Fallén, 1816)

***CHALCOSYRPHUS*** Curran, 1925

*Chalcosyrphus jacobsoni* (Stackelberg, 1921)

*Chalcosyrphus nemorum* (Fabricius, 1805)

*Chalcosyrphus nigripes* (Zetterstedt, 1838)

*Chalcosyrphus piger* (Fabricius, 1794)

*Chalcosyrphus rufipes* (Loew, 1873)

*Chalcosyrphus valgus* (Gmelin, 1790)

= *femoratus* auct. nec Linnaeus, 1758

***CRIORHINA*** Meigen, 1822

*Criorhina asilica* (Fallén, 1816)

***LEJOTA*** Rondani, 1857

*Lejota ruficornis* (Zetterstedt, 1843)

***SPHECOMYIA*** Latreille, 1829

*Sphecomyia vespiformis* Gorski, 1852

***SPILOMYIA*** Meigen, 1803

*Spilomyia diophthalma* (Linnaeus, 1758)

***SYRITTA*** Lepeletier & Serville, 1828

*Syritta pipiens* (Linnaeus, 1758)

***TEMNOSTOMA*** Lepeletier & Serville, 1828

*Temnostoma angustistriatum* Krivosheina, 2002

= *bombylans* auct. nec (Fabricius, 1805)

*Temnostoma apiforme* (Fabricius, 1794)

*Temnostoma carens* Gaunitz, 1936

*Temnostoma sericomyiaeforme* (Portschinsky, 1886)

*Temnostoma vespiforme* (Linnaeus, 1758)

***TROPIDIA*** Meigen, 1822

*Tropidia fasciata* Meigen, 1822

*Tropidia scita* (Harris, 1780)

***XYLOTA*** Meigen, 1822

*Xylota caeruleiventris* Zetterstedt, 1838

*Xylota florum* (Fabricius, 1805)

*Xylota ignava* (Panzer, 1798)

*Xylota jakutorum* Bagatshanova, 1980

= *caeruleiventris* auct. nec Zetterstedt, 1838

*Xylota meigeniana* Stackelberg, 1964

*Xylota segnis* (Linnaeus, 1758)

*Xylota suecica* (Ringdahl, 1943)

*Xylota sylvarum* (Linnaeus, 1758)

*Xylota tarda* Meigen, 1822

*Xylota triangularis* Zetterstedt, 1838

*Xylota xanthocnema* Collin, 1939

MICRODONTINAE Rondani, 1845

tribe Microdontini Rondani, 1845

***MICRODON*** Meigen, 1803

*Microdon analis* (Macquart, 1842)

= *eggeri* Mik, 1897

*Microdon miki* Doczkal & Schmid, 1999

= *latifrons* auct. nec (Loew, 1856)

*Microdon mutabilis* (Linnaeus, 1758)

## Excluded species

*Microdon devius* (Linnaeus, 1761) was not found within present borders.

*Neoascia annexa* (Müller, 1776) was included in the Finnish species list in Fauna Europaea (Speight 2004). Authors have not found any specimens or publications that reliably show *Neoascia annexa* to belong in the Finnish fauna.

*Paragus punctulatus* (Zetterstedt, 1838) was included in the Finnish species list in Fauna Europaea (Speight 2004). Authors have not found any specimens or publications that reliably show *Parasyrphus punctulatus* to belong in the Finnish fauna.

*Parasyrphus relictus* (Zetterstedt, 1838): The status of *Parasyrphus relictus* is unclear and the type material is lost (Speight 2013). *Parasyrphus relictus* was excluded from Finnish checklist by Haarto and Kerppola (2007b).

*Sphaerophoria* sp. B was discovered as a deformed specimen of *Sphaerophoria laurae* Goeldlin, 1989.

*Temnostoma meridionale* (Krivosheina & Mamayev, 1962) was included in the Finnish species list in Fauna Europaea (Speight 2004). Authors have not found any specimens or publications that reliably show *Temnostoma meridionale* to belong in the Finnish fauna.

## Notes

***Melanostoma mellinum* (Linnaeus, 1758)** and ***Melanostoma dubium* (Zetterstedt, 1837)** form a species complex and it is very doubtful whether present species concepts provide an accurate reflection of the number of *Melanostoma* species (Speight 2013).

***Platycheirus modestus* Ide, 1926**. A previously unpublished record. A new species to the Palaearctic. One male was collected in 4.7.2008 from *Obb*: Tornio (leg. I. Kakko).

***Dasysyrphus* spp.** Both *Dasysyrphus venustus* (Meigen, 1822) and *Dasysyrphus hilaris* (Zetterstedt, 1843) are known to contain several species (Haarto and Kerppola 2007a, Bartsch 2009a).

***Cheilosia barovskii* (Stackelberg, 1930)**. A previously unpublished record. Five females were collected in 30.5.1937 from *Ta*: Sääksmäki (leg. K.E. Kivirikko) and one female in 20.5.2013 from *N*: Sipoo (leg. T. Järveläinen).

***Myolepta nigra* (Loew, 1872)** was erroneously included in the list of Finnish flies (Hackman 1980) instead of *Helleniola nigra* (Zetterstedt, 1843) = *Chrysosyrphus niger* (Hackman 1982).

The used nomenclature of the species ***Eristalis nemorum* (Linnaeus, 1758)**, ***Eristalis arbustorum* (Linnaeus, 1758)** and ***Eristalis horticola* (De Geer, 1776)** follows the proposal in Case 3259 by Chandler et al. (2004) and the usage of these names is confirmed in Opinion 2153 by ICZN (International Commission on Zoological Nomeclature) (2006).

***Mallota tricolor* Loew, 1871**. A previously unpublished record. One male was collected in 4.8.1977 from *Ta*: Hämeenlinna (leg. M. Raekunnas) and another male in 13.6.–6.7.2013 from *Sa*: Joutseno (leg. J. Vilén).

***Microdon mutabilis* (Linnaeus, 1758)** and ***Microdon myrmicae* Schönrogge, Barr, Wardlaw, Napper, Gardner, Breen, Elmes & Thomas, 2002** form a species complex the species of which can be separate only by characters of the larvae and the puparia (Bartsch 2009b). In the Finnish checklist *Microdon mutabilis* represents either of the two species or include both of the species.
